# Change in the life expectancy gap among people with mental illness: a 14-year population-based cohort study in Australia

**DOI:** 10.1016/j.lanwpc.2026.101978

**Published:** 2026-09-18

**Authors:** Mike Trott, David Lawrence, Sean Halstead, Claudia Bull, Dan Siskind, Steve Kisely

**Affiliations:** a Faculty of Health, Medicine, and Behavioural Sciences, University of Queensland, Brisbane, Australia; b Metro South Addiction and Mental Health Services, Brisbane, Australia; c Queensland Centre for Mental Health Research, Wacol, Australia; d School of Population Health, Curtin University, Perth, Australia; e Deakin Lifespan Institute, School of Psychology, Faculty of Health, Deakin University, Burwood, VIC, Australia; f The ALIVE National Centre for Mental Health Research Translation, The University of Melbourne, Australia; g Departments of Psychiatry, Community Health and Epidemiology, Dalhousie University, Halifax, Nova Scotia, Canada

**Keywords:** Epidemiology, Mortality, Psychiatry, Mental health

## Abstract

**Background:**

People with psychiatric disorders die substantially earlier than the general population, with life expectancy gaps of 10–20 years reported internationally. Prior Australian data indicated this gap was widening, whereas evidence from other countries has been mixed. We aimed to determine whether the life expectancy gap in people with hospitalised psychiatric disorders versus the general population is changing in Queensland, Australia, and to identify which causes of death drive excess mortality.

**Methods:**

We conducted a retrospective population-based cohort study using linked Queensland hospital admissions and death registry data. Adults (≥18 years) with a principal psychiatric diagnosis across seven diagnostic categories were included, excluding dementia. Life expectancy was estimated using life-table methods and compared to Queensland norms. We calculated standardised mortality ratios (SMRs) across four time-periods and quantified the absolute and proportional contribution of specific causes to excess mortality using excess mortality rates per 100,000 person-years and attributable fractions.

**Findings:**

The analytic cohort comprised 255,448 individuals contributing 2,204,719 person-years. The life expectancy gap narrowed significantly for both sexes (p < 0·001): from 13·4 to 9·4 years in females and 16·7 to 12·1 years in males. All-cause SMRs declined from 2·35 (95% CI 2·23–2·47) to 1·73 (1·67–1·79) in females and 3·12 (2·98–3·25) to 2·10 (2·04–2·16) in males. The gap did not narrow significantly in either sex among people with schizophrenia or other psychoses, in females with affective psychoses, or in females with neurotic or alcohol and substance use disorders. Although intentional self-harm was the largest single contributor to proportional excess mortality in males, the combined excess mortality rate from physical health causes was substantially greater than from self-harm (440·0 and 441·9 versus 163·7 and 83·5 per 100,000 person-years in males and females respectively).

**Interpretation:**

The life expectancy gap between people with psychiatric disorders and the general population in Queensland has narrowed over 14 years, although the absolute gap remains considerable, and people with schizophrenia and related psychotic disorders have not shared equally in these gains. Physical illness is the principal driver of excess mortality, therefore prioritising physical health care for people with mental illness is essential to closing the gap, especially in psychotic disorders.

**Funding:**

None.

**Research in context**

**Evidence before this study**

We searched PubMed and Embase from inception to June 2026 for studies reporting life expectancy gaps, standardised mortality ratios, or years of potential life lost in people with psychiatric disorders, using terms related to life expectancy, premature mortality, and psychiatric or mental disorders, without language restriction. The foundational Australian evidence, from a retrospective analysis of Western Australian population registers covering 1985–2005, demonstrated that the life expectancy gap between people with psychiatric disorders and the general population widened over that period, largely because general population life expectancy rose faster than that of the psychiatric cohort. International evidence has been mixed: Scandinavian register-based cohort studies have shown a modest narrowing of the mortality gap, whereas a recent meta-analysis found no significant reduction in years of potential life lost over time for most disorders, and a pooled analysis of mortality risk ratios suggested the relative gap may be widening. Cause-of-death analyses consistently demonstrate that, while intentional self-harm contributes disproportionately to years of potential life lost, most excess deaths in absolute terms arise from physical health conditions, particularly cardiovascular disease and cancer. We found no Australian study examining temporal trends in the life expectancy gap since the Western Australian analysis, leaving it unknown whether the subsequent decades of mental health reform, physical health co-management initiatives, and improved cancer and cardiac care has altered this trajectory.

**Added value of this study**

This study provides the first updated, population-level evidence on life expectancy trends in people with hospitalised psychiatric disorders in Australia, covering 14 years (2009–2023) across 255,448 individuals and seven psychiatric diagnostic categories. We demonstrate that the life expectancy gap has narrowed significantly overall in both males and females, providing more optimistic evidence than most of the international literature. This improvement, however, was not equally shared across diagnostic categories: people with schizophrenia and related psychotic disorders showed no significant narrowing in either sex, identifying a persistent and deepening inequity within the psychiatric population. We additionally provide cause-specific excess mortality data demonstrating that physical health conditions account for approximately twice (males) to three times (females) the absolute excess mortality burden of intentional self-harm, reinforcing the primacy of physical health as a target for intervention.

**Implications of all the available evidence**

Taken together, the evidence suggests that broader improvements in population health may contribute to narrowing mortality disparities for people with psychiatric disorders, but these gains are not reaching those with the most severe diagnoses. The persistent, unnarrowed life expectancy gap in schizophrenia and related psychoses demands specific clinical and policy attention, including investment in physical health monitoring, metabolic risk management, and equitable access to cancer and cardiovascular care. The dominance of physical health causes across all diagnostic groups in absolute excess mortality should inform resource allocation decisions at both service and health system levels, and underscores the need for integrated physical and mental health care as a matter of routine practice.

## Introduction

The excess mortality associated with mental illness has been extensively documented.^1^^,^^2^ In a meta-analysis of approximately 150 studies, people with mental illness had more than double the risk of all-cause mortality compared to those without mental disorder.^3^ This applied to a broad range of diagnoses, including psychotic disorders (e.g. schizophrenia spectrum disorders and affective psychoses), neurotic disorders, stress/adjustment disorders and depressive disorders, with the highest rates found in psychotic and substance use disorders.^1^^,^^3^, ^4^, ^5^ Although much of the attention has focussed on the elevated risks of suicide,^6^ most of the excess mortality risk has been reported to be due to physical illnesses, such as cardiovascular diseases, respiratory diseases and cancer as opposed to unnatural causes including suicide and accidents.^4^^,^^5^^,^^7^, ^8^, ^9^

Excess mortality in people with mental illness is often expressed using standardised mortality rates or mortality rate ratios. However, because mortality varies by age at onset and time since diagnosis, these ratios have limitations.^5^^,^^10^ In particular, differences in cohort composition and length of follow-up can influence the outcome. Systematic reviews have therefore increasingly reframed the issue in terms of life expectancy.^3^^,^^5^^,^^11^^,^^12^ Because this outcome is calculated by cumulating across all ages, life expectancy can reflect changes in mortality rates across ages. In particular, these measures capture the effect on survival of conditions that lead to excess mortality at younger ages. In a recent meta-analysis of over 12 million individuals, people with mental illness died nearly 15 years earlier than the general population, with substance use disorders associated with the lowest life expectancy, followed by schizophrenia-spectrum disorders.^5^

Several systematic reviews and narrative syntheses have also examined whether the mortality gap has changed over time, with conflicting results. While increased mortality rates in people with schizophrenia have remained largely unchanged over time and may have widened in some settings,^3^^,^^4^^,^^13^ others have suggested a reduction in the mortality gap compared to the general population,^4^ while others have suggested it has remained the same.^14^ A further systematic review found higher mortality rates in more recent studies, particularly those commencing in the 1990s compared to those before 1970.^3^ These results were mirrored in a meta-regression of life expectancy spanning three decades (pre-2001, 2001–2010, and post-2010) where the longevity gap persisted for most disorders across all three periods.^5^

There are several difficulties in interpreting the results of existing systematic reviews in this area. The first is that data for individual diagnoses generally come from different studies with variations in methods and settings resulting in substantial statistical heterogeneity across studies.^3^, ^4^, ^5^^,^^14^ Another is that some report mortality rates while others use measures of longevity. One study that investigated a wide range of psychiatric disorders over 20 years in the same population was from Western Australia.^15^ This reported a widening gap in life expectancy from 1985 to 2005, the differential being particularly marked for people with substance use disorders and schizophrenia. However, these data are dated, and it remains unknown whether this gap continues to change across time. The aim of this study, therefore, was to compare mortality in people with hospitalised mental illness to that of the general population in another Australian state (Queensland).

## Methods

Ethical approval was obtained from the Metro South Human Research Ethics Committee (HREC/2025/QMS/121215). Reporting of this study followed STrengthening the Reporting of OBservational studies in Epidemiology (STROBE) guidance,^16^ and the REporting of studies Conducted using Observational Routinely-collected health Data (RECORD) Statement.^17^

### Patient and public involvement

Patients and members of the public were not involved in the design, conduct, reporting, or dissemination plans of this research.

### Study design and setting

This was a population-based retrospective cohort study using linked administrative health and mortality data from Queensland, Australia.

### Data sources

Hospital admissions data were obtained from the Queensland Hospital Admitted Patient Data Collection (QHAPDC), which records all inpatient episodes in public hospitals across Queensland, including International Classification of Diseases, 10th Revision—Australian Modification (ICD-10-AM) diagnosis codes per episode. Death data were obtained from the Queensland Registry of Births, Deaths and Marriages, linked to the hospital admissions data by the Data Services Branch (DSB) of Queensland Health. Information on the accuracy and quality of data linkage is available via the Queensland Data Linkage Framework.^18^ Comparative Queensland general population life tables and mortality rates were sourced from the Australian Bureau of Statistics (ABS) Life Tables, covering the periods 2009–2011, 2012–2014, 2015–2017, 2018–2020, and 2021–2023.^19^

### Study cohort

The initial cohort included all individuals aged 18 years or older who had at least one hospital admission with a principal or secondary mental health diagnosis (ICD-10-AM Chapter F) in Queensland between 1 January 2000 and 31 December 2023. The observation window for the current analysis was restricted to 1 January 2009–31 December 2023 to align with available ABS life table periods (Fig. 1), and further restricted to individuals with a principal F-Chapter diagnosis. Individuals with any recorded diagnosis of dementia (ICD-10-AM codes F00–F03, G30) at any point were excluded, given the substantially different epidemiology of mortality in this population.^15^ Cohort entry was defined as the date of the first hospital admission with a mental health diagnosis within the study window. For individuals with their first mental health admission prior to 2009, follow-up was left truncated to begin from 1 January 2009. Follow-up continued until death, or 31 December 2023, whichever occurred first.

**Fig. 1:**
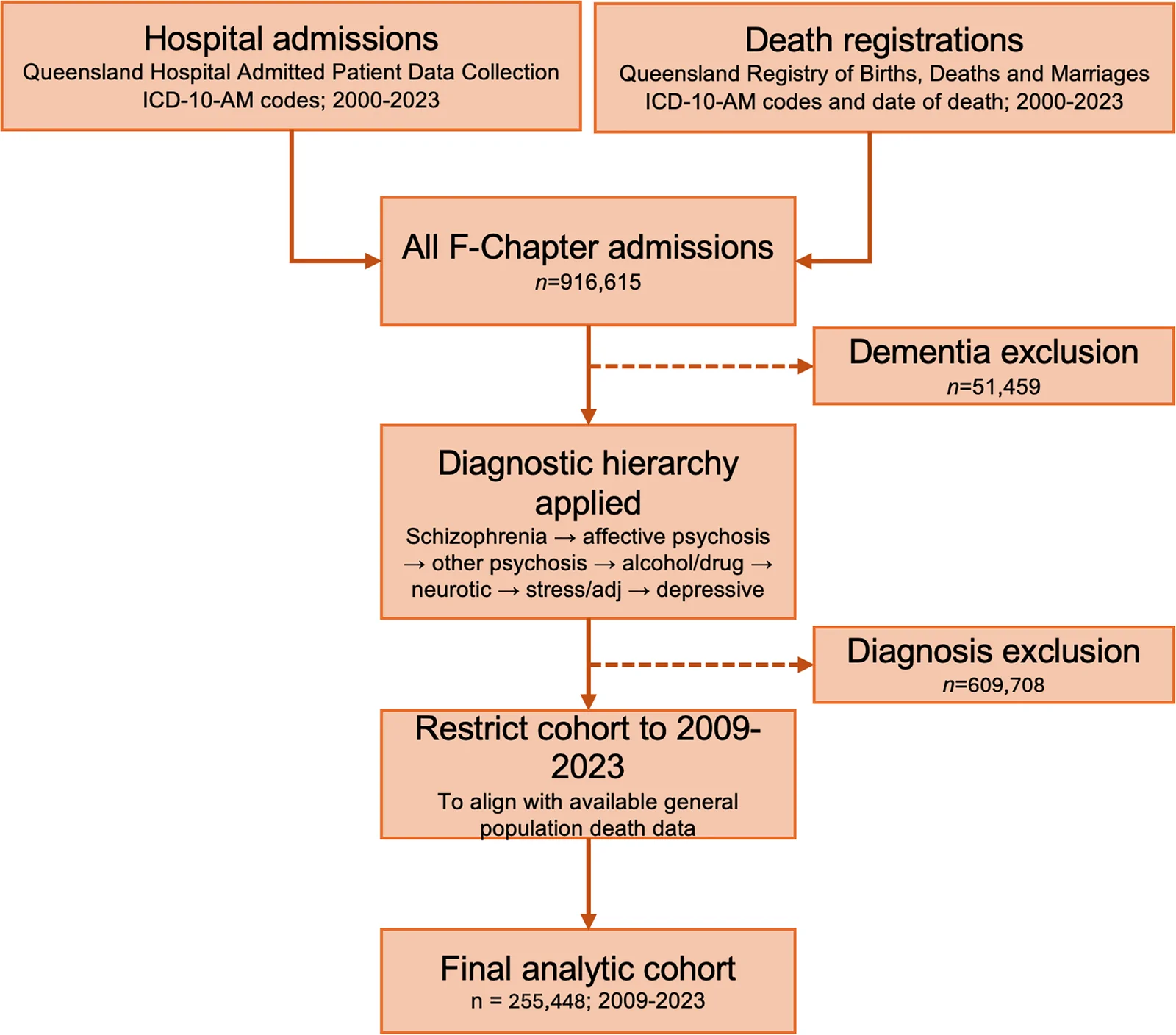
Cohort creation.

### Principal psychiatric diagnosis

Each person was assigned a principal psychiatric diagnosis using a hierarchical approach based on principal diagnosis codes.^15^^,^^20^^,^^21^ Diagnoses were grouped into seven categories according to the following hierarchy:

1. Schizophrenia (F20);
2. Affective psychoses (F30, F31; F32·3; F33·3);
3. Other psychoses (F21–F25, F28–F29);
4. Alcohol or substance use disorders (F10–F19);
5. Neurotic disorders (F40–F42, F44–F45, F48);
6. Stress and adjustment reactions (F43); and
7. Depressive disorders (F32–F34, F38–F39, excluding cases with psychotic episodes).

Mental health diagnoses recorded across the entire observation period (2000–2023) were considered. This approach gives precedence to more severe disorders such as psychosis and treats other conditions such as substance use disorders as potential comorbidities when a more severe diagnosis is also present.

### Calendar periods

The analysis window was divided into 5 non-overlapping 3 years calendar periods: 2009–2011, 2012–2014, 2015–2017, 2018–2020, and 2021–2023, consistent with the ABS life table reference periods.^19^

### Life expectancy estimation

Abridged period life tables were constructed for each diagnostic group, sex, and calendar period (as well as the entire psychiatric cohort comprising all individuals in the diagnostic hierarchy combined) using the Chiang method,^22^ with a radix of 100,000 and a midpoint person-years assumption for all 5 years age intervals.^23^ Life expectancy at age 18 was extracted from each life table as the primary outcome. Abridged life tables were constructed for each combination of principal psychiatric diagnosis, sex, and calendar period, using 5 years age bands from 18 years onwards. Person-years at risk were derived by Lexis expansion.^24^ Each person's follow-up was split across calendar periods and 5 years age bands, with deaths assigned to the period and age band in which they occurred. All-cause mortality rates were calculated as deaths divided by person-years within each cell. A three-point moving average was applied to smooth age-specific mortality rates before constructing the life tables,^25^ with the aim to reduce the instability that would arise when age-, sex-, diagnosis-, and period-specific cells contain few deaths, causing adjacent age bands to fluctuate. Life expectancy at age 18 was estimated from the resulting abridged life tables using the Chiang actuarial method.^26^ 95% confidence intervals were derived by parametric bootstrapping using Poisson-distributed random draws across 500 iterations. The life expectancy gap was calculated as the difference between the Queensland general population at age 18 (from ABS life tables) and the cohort within each period and sex stratum. Standardised mortality ratios (SMRs) were calculated for each diagnostic group, sex, and calendar period as the ratio of observed deaths to expected deaths, where expected deaths were derived by applying Queensland general population age-sex-specific mortality rates to the person-years contributed by the cohort within each stratum, with Poisson 95% confidence intervals for SMRs calculated using the chi-squared method.^27^

### Causes of death

Cause of death was classified using ICD-10-AM codes, grouped into the following categories^15^: coronary heart disease, cerebrovascular disease, all cancers, Chronic Obstructive Pulmonary Disease (COPD), influenza and pneumonia, diabetes, liver disease, chronic kidney disease, dementia (including Alzheimer's), intentional self-harm (resulting in suicide), accidental poisoning, and assault, full information regarding coding can be found in Supplementary Table S1. We also created a composite physical health category, summing all cause-specific deaths except intentional self-harm and assault. Cause-specific death rates per 100,000 person-years and proportional distributions were calculated by diagnostic group, sex, and calendar period. Expected deaths by cause were calculated by applying age, sex-, and time-specific Australian national mortality rates from the General Record of Incidence of Mortality (GRIM) books^28^ to cohort person-years within each stratum. Excess deaths by cause were calculated as the difference between observed and expected deaths, and expressed both as an absolute excess mortality rate (excess deaths per 100,000 person-years) and as the proportional contribution of each cause to total excess mortality. We also calculated the attributable fraction of the exposed (AFe) by dividing excess deaths by observed deaths. To assess the potential influence of COVID-19 on cause-specific findings, a sensitivity analysis was performed comparing pre (2009–2020) and post (2021–2023) time periods.

### Statistical analysis

All analyses were conducted using RStudio (Version 4·4·0). Temporal trends in life expectancy, life expectancy gaps, and SMRs were assessed using linear regression, with calendar period treated as an ordinal variable. All trend tests were conducted separately for each diagnostic group and sex. To assess whether temporal changes in the diagnostic composition of the cohort account for any changes in the combined (all-diagnoses) life expectancy gap, we conducted a sensitivity analysis in which the all-diagnoses gap was recomputed with the diagnostic composition fixed at the pooled whole-period distribution, with age-specific mortality rates for each diagnostic group reweighted by these fixed weights before constructing life tables.

## Results

The linked dataset comprised 916,615 individuals that had been admitted to hospital with an ICD-10-AM F Chapter diagnosis, of whom 51,459 (5·6%) were excluded due to dementia diagnoses. 609,708 people were excluded as they either did not have a principal diagnosis or their principal diagnosis fell outside of the seven diagnostic categories. The final analytic cohort included 255,448 individuals contributing person-time between 1 January 2009 and 31 December 2023, see Fig. 1. Median follow-up was 8·6 years (IQR 4·0–14·6), during which 28,764 deaths were observed, corresponding to 2,204,719 person-years of follow-up.

The mean (SD) age at cohort entry was 41·5 (±16·8) years, and 52·1% were male. The most common diagnostic groups were substance use disorders (30·0%), depressive disorders (16·2%), and neurotic disorders (15·6%), see Table 1. Demographic characteristics by period were stable across calendar periods, see Supplementary Table S2. The diagnostic composition of the cohort shifted over the study period, with the proportion of entrants with schizophrenia and affective psychosis declining and lower-mortality groups such as neurotic disorders increasing (Supplementary Tables S3 and S4).

**Table 1:** Demographic cohort information.

Principal diagnoses	Males				Females			
	Total *n*	Deaths *n*	PY	Mortality rate	Total *n*	Deaths *n*	PY	Mortality rate
Psychiatric cohort	133,113	16,463	1,151,149	14·3	122,335	12,301	1,053,570	11·7
Schizophrenia	18,017	2040	196,718	10·4	9388	1215	101,319	12·0
Affective psychoses	8773	996	83,970	11·9	12,728	1180	125,654	9·4
Other psychotic disorders	7444	760	61,779	12·3	6705	630	55,110	11·4
Alcohol or substance use disorders	48,106	6754	396,623	17·0	29,342	2897	249,462	11·6
Neurotic disorders	13,638	2029	98,271	20·6	25,915	3245	192,292	16·9
Stress and adjustment disorders	17,985	1590	160,009	9·9	16,208	927	146,292	6·3
Depressive disorders	19,150	2294	153,778	14·9	22,049	2207	183,440	12·0

Mortality rate calculated as deaths per 1000 person years; PY = person years.

### Life expectancy

Life expectancy in the entire cohort increased from 71·5 years (95% CI 70·9–72·2) in 2009–2011 to 76·1 years (95% CI 75·7–76·4) in 2021–2023 in females, and from 64·2 years (95% CI 63·7–64·6) in 2009–2011 to 69·3 years (95% CI 68·9–69·7) in 2021–2023 in males. Over the same period, life expectancy in the general Queensland population increased from 84·6 to 85·3 years in females, and from 80·2 to 81·1 years in males. The life expectancy gap significantly decreased across the study period in both males and females (p < 0·001 and p = 0·001 respectively), from 16·7 to 12·1 years in males and from 13·4 to 9·4 years in females, see Fig. 2. Diagnostic composition sensitivity analyses showed similar results in terms of both magnitude and significance, see Supplementary Table S5. Most diagnostic groups showed significant decreasing life expectancy gaps. Exceptions were females with affective psychosis (p = 0·182), males and females with other psychotic disorders (p = 0·091 and p = 0·152 respectively), females with neurotic disorders (p = 0·052), females with alcohol or substance use disorders (p = 0·050), and both males and females with schizophrenia (males p = 0·309, females p = 0·113), whose gaps showed no statistically significant change across the study period, see Table 2 and Fig. 2.

**Fig. 2:**
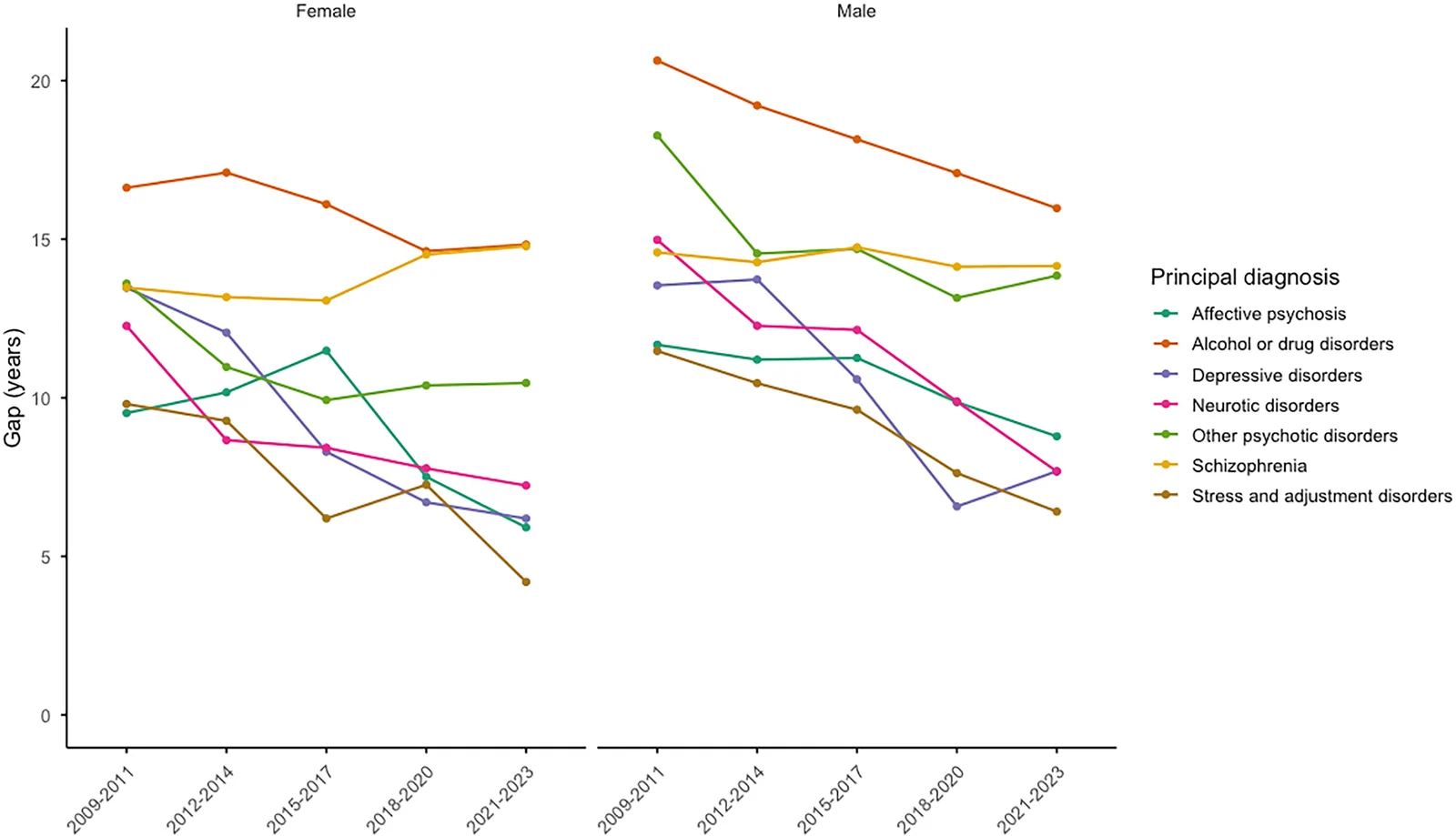
Life expectancy gap by sex and principal diagnosis.

**Table 2:** Life expectancy gap (in years) across time stratified by sex and principal psychiatric diagnosis.

Principal diagnosis	Sex	Years					p-value^a^
		2009–2011	2012–2014	2015–2017	2018–2020	2021–2023	
Psychiatric cohort	Male	**16·7**	**15·3**	**14·5**	**12·8**	**12·1**	**<0·001**
	Female	**13·4**	**12·3**	**11·3**	**10·3**	**9·4**	**<0·001**
Schizophrenia	Male	14·6	14·3	14·7	14·1	14·2	0·309
	Female	13·5	13·2	13·1	14·5	14·8	0·113
Affective psychosis	Male	11·7	11·2	11·3	9·9	8·8	0·019
	Female	9·5	10·2	11·5	7·5	5·9	0·182
Other psychotic disorders	Male	18·3	14·5	14·7	13·1	13·9	0·091
	Female	13·6	11·0	9·9	10·4	10·5	0·152
Alcohol or substance use disorders	Male	**20·6**	**19·2**	**18·1**	**17·1**	**16·0**	**<0·001**
	Female	16·6	17·1	16·1	14·6	14·8	0·050
Neurotic disorders	Male	**15·0**	**12·3**	**12·1**	**9·9**	**7·7**	**0·004**
	Female	12·3	8·7	8·4	7·8	7·2	0·052
Stress and adjustment disorders	Male	**11·5**	**10·5**	**9·6**	**7·6**	**6·4**	**0·001**
	Female	**9·8**	**9·3**	**6·2**	**7·3**	**4·2**	**0·031**
Depressive disorders	Male	**13·5**	**13·7**	**10·6**	**6·6**	**7·7**	**0·032**
	Female	**13·5**	**12·1**	**8·3**	**6·7**	**6·2**	**0·007**

Bold columns indicate statistically significant results where p = <0·05.

a p-values represent linear trends over time.

### Standardised mortality ratios

Overall mortality in the psychiatric cohort relative to the general population declined over time from SMR 2·35 (95% CI 2·23–2·47) in 2009–2011 to 1·73 (95% CI 1·68–1·79) in 2021–2023 for females, and from 3·12 (95% CI 2·98–3·25) in 2009–2011 to 2·10 (95% CI 2·04–2·16) in 2021–2023 for males, with a significant temporal trend towards lower SMRs in both sexes (p < 0·001 for both). Most diagnostic groups showed significantly decreasing SMRs across the study period. Exceptions were females with affective psychosis (p = 0·070), males and females with other psychotic disorders (p = 0·063 and p = 0·490 respectively), females with stress and adjustment disorders (p = 0·062), and females with schizophrenia (p = 0·557), see Table 3 and Supplementary Figure S1.

**Table 3:** Standardised mortality ratios (in years) across time stratified by sex and principal psychiatric diagnosis.

Principal diagnosis	Sex	Years					p-value^a^
		2009–2011	2012–2014	2015–2017	2018–2020	2021–2023	
Psychiatric cohort	Male	**3·12 (2·98**–**3·25)**	**2·83 (2·72**–**2·94)**	**2·53 (2·44**–**2·62)**	**2·25 (2·18**–**2·32)**	**2·10 (2·04**–**2·16)**	**<0·001**
	Female	**2·35 (2·23**–**2·47)**	**2·13 (2·04**–**2·23)**	**2·01 (1·94**–**2·10)**	**1·91 (1·84**–**1·98)**	**1·73 (1·68**–**1·79)**	**<0·001**
Schizophrenia	Male	**3·88 (3·43**–**4·38)**	**3·63 (3·24**–**4·05)**	**3·71 (3·37**–**4·07)**	**3·30 (3·01**–**3·62)**	**3·26 (2·99**–**3·54)**	**0·022**
	Female	3·17 (2·68–3·72)	2·87 (2·47–3·32)	2·71 (2·37–3·08)	3·10 (2·76–3·47)	2·84 (2·55–3·15)	0·557
Affective psychosis	Male	**2·24 (1·87**–**2·66)**	**2·19 (1·87**–**2·55)**	**2·07 (1·79**–**2·38)**	**1·97 (1·72**–**2·24)**	**1·77 (1·56**–**2·00)**	**0·006**
	Female	2·21 (1·88–2·59)	2·23 (1·93–2·56)	2·28 (2·02–2·57)	1·90 (1·67–2·14)	1·60 (1·42–1·79)	0·070
Other psychotic disorders	Male	3·54 (2·89–4·30)	2·75 (2·26–3·32)	2·56 (2·16–3·02)	2·42 (2·07–2·81)	2·48 (2·18–2·82)	0·063
	Female	2·05 (1·62–2·55)	1·85 (1·50–2·27)	1·79 (1·49–2·15)	1·86 (1·56–2·19)	1·92 (1·66–2·20)	0·490
Alcohol or substance use disorders	Male	**4·50 (4·21**–**4·81)**	**3·95 (3·71**–**4·19)**	**3·55 (3·36**–**3·75)**	**3·27 (3·11**–**3·43)**	**3·00 (2·86**–**3·14)**	**<0·001**
	Female	**4·34 (3·87**–**4·85)**	**4·01 (3·65**–**4·41)**	**3·65 (3·36**–**3·96)**	**3·24 (3·00**–**3·49)**	**3·07 (2·87**–**3·28)**	**<0·001**
Neurotic disorders	Male	**2·41 (2·13**–**2·73)**	**2·00 (1·79**–**2·24)**	**1·85 (1·68**–**2·04)**	**1·67 (1·52**–**1·83)**	**1·50 (1·38**–**1·64)**	**0·001**
	Female	**1·78 (1·61**–**1·98)**	**1·53 (1·40**–**1·67)**	**1·56 (1·45**–**1·68)**	**1·49 (1·39**–**1·60)**	**1·33 (1·25**–**1·42)**	**0·027**
Stress and adjustment disorders	Male	**2·10 (1·82**–**2·43)**	**1·99 (1·76**–**2·25)**	**1·77 (1·58**–**1·98)**	**1·52 (1·37**–**1·69)**	**1·37 (1·25**–**1·50)**	**0·001**
	Female	2·03 (1·67–2·45)	2·08 (1·77–2·44)	1·61 (1·37–1·87)	1·78 (1·56–2·02)	1·40 (1·23–1·58)	0·062
Depressive disorders	Male	**2·28 (2·03**–**2·54)**	**2·37 (2·15**–**2·60)**	**1·76 (1·60**–**1·93)**	**1·41 (1·28**–**1·54)**	**1·44 (1·33**–**1·56)**	**0·025**
	Female	**2·18 (1·95**–**2·43)**	**1·88 (1·70**–**2·08)**	**1·65 (1·50**–**1·81)**	**1·46 (1·33**–**1·59)**	**1·32 (1·22**–**1·44)**	**<0·001**

Bold columns indicate statistically significant results where p = <0·05.

a p-values represent linear trends over time.

### Causes of death

7837 deaths could not be classified into the pre-specified cause-of-death categories and were therefore excluded from the cause-specific analysis. Regarding absolute mortality rates, physical health conditions accounted for most deaths, with all cancers representing the highest cause-specific mortality rate in both sexes (289·2 per 100,000 person-years in males, 231·5 in females). Coronary heart disease was the second highest contributor in females (125·5 per 100,000 person-years), whereas self-harm was second in males (187·9 per 100,000 person-years). These were also among the largest causes of death in proportional terms (cancers 20·0% of male deaths and 19·5% of female deaths; coronary heart disease 11·9% and 10·8% respectively). When mortality was expressed relative to the general population, intentional self-harm was the single largest contributor to excess mortality in males (excess mortality rate 163·7 per 100,000 person-years), whereas in females coronary heart disease was the leading contributor (101·7 per 100,000 person-years), with intentional self-harm third. The combined excess mortality rate from physical health causes (excluding intentional self-harm, assault, and accidental poisoning) was 440·0 per 100,000 person-years in males and 441·9 in females, approximately 2·7 times the self-harm rate in males and 5·3 times in females, see Fig. 3. In males, the leading contributors to excess mortality after intentional self-harm were cancer (112·9 per 100,000 person-years), coronary heart disease (109·1), accidental poisoning (83·7), and liver disease (76·8); in females, coronary heart disease was followed by cancer (89·4), intentional self-harm (83·5), and chronic obstructive pulmonary disease (56·5). These patterns varied substantially by diagnostic group, see Table 4 and Supplementary Table S6. Sensitivity analyses showed no substantial differences between pre- and post-COVID time periods, see Supplementary Figure S2.

**Fig. 3:**
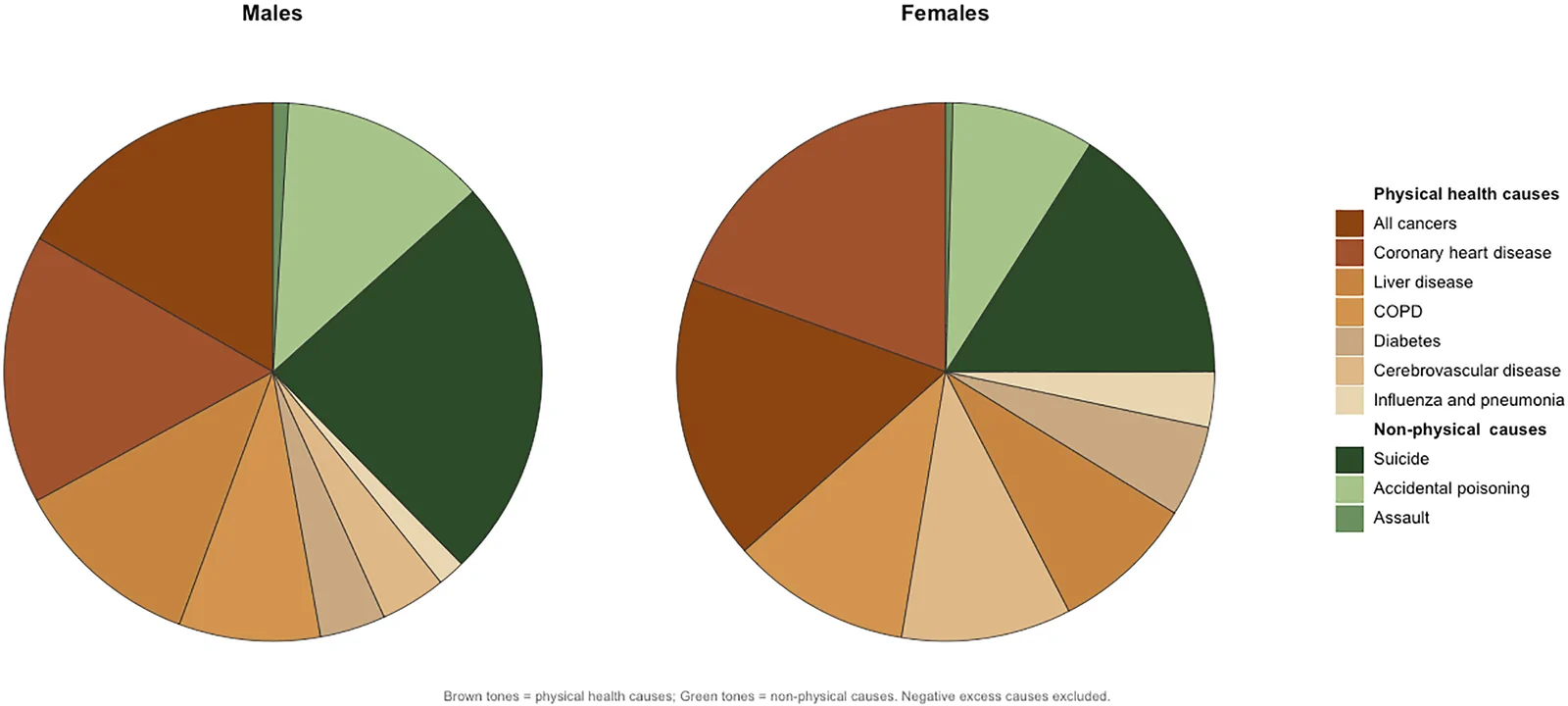
Excess mortality rates by cause of death.

**Table 4:** Excess deaths in people with mental illness stratified by sex and cause of death.

	Observed deaths	Expected deaths	Excess deaths	Mortality rate (per 100,000 PY)	Excess mortality rate (per 100,000 PY)	Afe (%)
Males						
All physical health causes	8819	3753·4	5065·6	766·1	440·0	57·4
Intentional self-harm	2163	278·8	1879·7	187·9	163·7	87·1
All cancers	3329	2029·8	1299·2	289·2	112·9	39·0
Coronary heart disease	1936	680·0	1256·0	168·2	109·1	64·9
Accidental poisoning	1099	135·7	963·3	95·5	83·7	87·7
Liver disease	1035	150·8	884·2	89·9	76·8	85·4
COPD	879	220·7	658·3	76·4	57·2	74·9
Diabetes mellitus	481	175·1	305·9	41·8	26·6	63·6
Cerebrovascular disease	517	211·4	305·6	44·9	26·5	59·1
Dementia incl· Alzheimer's	331	141·4	189·6	28·8	16·5	57·3
Influenza and pneumonia	177	49·5	127·5	15·4	11·1	72·0
Assault	91	20·3	70·7	7·9	6·1	77·7
Chronic kidney disease	134	94·7	39·3	11·6	3·4	29·3
Females						
All physical health causes	7243	2587·7	4655·3	687·5	441·9	64·3
Coronary heart disease	1322	250·4	1071·6	125·5	101·7	81·1
All cancers	2439	1497·4	941·6	231·5	89·4	38·6
Intentional self-harm	957	77·3	879·7	90·8	83·5	91·9
COPD	774	178·6	595·4	73·5	56·5	76·9
Cerebrovascular disease	754	191·6	562·4	71·6	53·4	74·6
Liver disease	545	70·1	474·9	51·7	45·1	87·1
Accidental poisoning	524	50·9	473·1	49·7	44·9	90·3
Dementia incl· Alzheimer's	618	178·6	439·4	58·7	41·7	71·1
Diabetes mellitus	409	107·7	301·3	38·8	28·6	73·7
Influenza and pneumonia	221	38·3	182·7	21·0	17·3	82·7
Chronic kidney disease	161	75·0	86·0	15·3	8·2	53·4
Assault	31	7·6	23·4	2·9	2·2	75·5

AFe = attributable fraction; COPD = Chronic Obstructive Pulmonary Disease.

SMRs declined significantly across most causes of death in both sexes, with significant reductions observed for intentional self-harm (−9·8% per period in males, p = 0·005, −12·5% per period in females, p = 0·002), with significant declines in cancer, coronary heart disease, COPD, diabetes, and liver disease in males, and liver disease, influenza and pneumonia, and cancer in females. Full information can be found in Supplementary Table S7.

## Discussion

This retrospective cohort study examined trends in life expectancy and excess mortality among people with hospitalised psychiatric disorders in Queensland, Australia over a 14 years period from 2009 to 2023. The principal finding was that the life expectancy gap between people with mental illness and the general Queensland population narrowed significantly over this period for most diagnostic groups. Despite this improvement, the absolute gap still exceeds 12 years for males and 10 years for females across all mental health disorders combined, underscoring that people with mental illness continue to die considerably younger than the general population. Our findings also emphasise that these adverse outcomes are most marked for people with schizophrenia and related psychotic disorders.

The narrowing we observed appears to be driven primarily by improvements in the psychiatric cohort life expectancy rather than deterioration in the general population, which increased by only around one year over the same period. The narrowing therefore reflects genuine relative improvements in the psychiatric cohort survival and cannot be attributed to the comparison population becoming less healthy. Furthermore, although the diagnostic composition changed during the study period, sensitivity analyses showed this did not affect the magnitude or significance of the primary results. This pattern suggests that people with mental illness in Queensland have experienced disproportionate gains in survival relative to the general population across this period, though they have not yet closed the gap to anything approaching parity. Our findings contrast with the most recent systematic review where gaps in life expectancy persisted over time in stratified analyses by study period (pre-2001, 2001–2010, and post-2010).^5^ A possible explanation is that the use of such broad time intervals in that review disguised trends that might have become more apparent with analysis by every 3 years as in the current study. It is also likely that because our hospital-ascertained cohort is more clinically severe than community-inclusive samples (and absolute gaps larger), differences from studies incorporating community-managed cases may reflect case-mix rather than genuinely different mortality estimates.

There were reductions in potentially avoidable deaths for all psychiatric diagnoses except schizophrenia and related psychotic disorders. For example, SMRs for alcohol and substance use disorders were higher than those for schizophrenia in 2009–2011 but then declined to values comparable to other non-psychotic disorders by 2021–2023. Although the gap narrowed most steeply for alcohol and substance use disorders, the burden in this group remained the highest of any diagnostic category, concurrent with previous mortality studies.^29^^,^^30^ The improving trend, therefore, should not mask the high mortality these patients continue to experience, and further interventional and public health research is warranted to reduce this burden. By contrast, the life expectancy gap did not narrow significantly for either sex with schizophrenia, or for females with affective psychosis or either sex with other psychoses. Notably, while males with affective psychosis showed a significant narrowing in both the life expectancy gap and SMR, period-specific mortality rates suggest that the absolute mortality rate in this group remained largely unchanged across the study period. The narrowing life expectancy gap in males with affective psychosis therefore appears to reflect improvements in general population life expectancy rather than reductions in cohort mortality. In females with affective psychosis, a transient increase in mortality rates in 2015–2017 contributed to the non-significant and non-linear trend in the life expectancy gap, the cause of which is unclear and warrants further investigation.

In males and females with stress and adjustment disorders, absolute life expectancy gaps narrowed significantly, however in females there was no significant change in their SMRs. Interestingly Lawrence et al. reported that females with stress and adjustment disorders were the only group without a significant life expectancy gap compared to the general population, however they also reported that the gap was widening in this population, contrary to our findings.^15^ Our finding of a narrowing absolute gap two decades later may indicate that the cohort survival has begun to improve at a rate comparable to the general population, consistent with a SMR. Furthermore, the disparate results in this population across studies may be due to substantial heterogeneity of conditions in this diagnostic category. Although beyond the scope of this study, future studies should aim to disaggregate this category by condition (e.g. post-traumatic stress disorders only) to determine granular trends.

Reductions in excess deaths were observed for intentional self-harm, cancers, coronary heart disease, COPD, diabetes, and liver disease in males, and for intentional self-harm, liver disease, influenza and pneumonia, and cancer in females. These improvements likely reflect a combination of advances in the treatment of physical health conditions, greater attention to physical health monitoring in psychiatric populations, and improved access to care over this period. Notably, coronary heart disease SMRs showed no significant improvement in females, which warrants further investigation.

Several mechanisms may explain the persistent mortality risk in people with mental illness, especially psychotic disorders. These include social disadvantage, individual vulnerabilities, substance use and lifestyle, as well as structural issues such as fragmented health care and barriers to accessing services.^31^ The mechanistic pathways resulting in premature mortality, however, differ substantively across diagnostic groups. In people with alcohol and substance use disorders, for example, excess mortality was dominated by the direct physical sequelae of substance use, most notably liver disease, whereas in schizophrenia the excess was concentrated in cardiovascular disease, consistent with the metabolic consequences of the illness and its treatment. These distinct causal pathways suggest, therefore, that single interventions are unlikely to serve the whole cohort, and that prevention must be tailored to the dominant mechanism in each group.

In absolute terms, cancers and coronary heart disease were the leading causes of death. Although intentional self-harm had the highest excess mortality rate of any individual cause, the combined excess mortality rate from physical health causes was approximately twice as large as intentional self-harm in males and nearly three times as large in females, underscoring that physical illness represents the dominant contributor to premature mortality in this population.^4^^,^^5^^,^^7^, ^8^, ^9^ Nonetheless, the suicide burden was considerable, with intentional self-harm the second most common cause of death in men and the single largest contributor to excess mortality in both sexes, and was especially pronounced in men with psychotic disorders. This is in agreement with previous reviews reporting that suicide and self-harm are among the highest causes of premature mortality in people with psychotic disorders and schizophrenia specifically.^15^^,^^32^ Meta-analytic evidence has shown that this group is more likely to die by jumping from heights and drowning, which has been hypothesised to be due to severe psychotic phenomenology (e.g. command hallucinations).^33^ Lethal means counselling, along with other suicide prevention strategies, should be a priority in this population. Among physical causes, coronary heart disease was the leading contributor to excess mortality, followed by liver disease and cancers. The prominence of cardiovascular disease as one of the leading contributors to excess physical health mortality is broadly consistent with the earlier Western Australian study,^15^ as well as several systematic reviews of this area.^4^^,^^5^^,^^7^, ^8^, ^9^ Cardiovascular mortality was particularly elevated in people with schizophrenia, with coronary heart disease accounting for a substantial proportion of excess deaths, likely reflecting both metabolic risks and higher baseline cardiovascular risk factors in this population.^34^ In addition, there are the adverse effects of antipsychotic medications, including weight gain, dyslipidaemia, and impaired glucose tolerance.^34^, ^35^, ^36^ The growing use of second-generation antipsychotics may have paradoxically increased cardiometabolic risk in some individuals despite improving psychiatric symptom control.^34^^,^^36^

Despite this elevated burden of physical illness, significant disparities exist in access to and quality of healthcare for people with mental illness, including reduced access to cardioprotective medications, lower participation in cancer screening programmes, and reduced likelihood of receiving guideline-concordant treatments.^2^^,^^37^, ^38^, ^39^, ^40^, ^41^, ^42^, ^43^, ^44^ These inequities are likely to contribute to the excess mortality from cardiovascular disease and cancer observed in this cohort. We also found no substantial differences in findings pre- and post-COVID-19, which may reflect the largely maintained continuity of mental health care in Queensland during the pandemic, including a shift to telepsychiatry without significant reductions in antipsychotic prescribing.^45^^,^^46^

This study has several important strengths. The use of data covering the full Queensland population reduces selection bias and provides sufficient statistical power to examine trends within diagnostic subgroups, as well as being representative at the population level. Several limitations should also be acknowledged. First, the cohort was restricted to individuals with hospital-based psychiatric admissions and therefore does not capture people managed exclusively in primary care or community settings, who may have different mortality outcomes. This is likely to result in a more clinically severe cohort, which may partly explain the larger absolute gaps observed compared with some international studies. Furthermore, we could not assess whether changes in hospitalisation practice altered the diagnostic characteristics of those admitted over time; if higher-risk individuals became less likely to be hospitalised, for example, this could contribute to the observed narrowing independently of any genuine improvement in survival.

Second, the general population comparator derived from ABS life tables also inherently includes people with mental illness, which will modestly attenuate the observed gap. Third, the cause-of-death analyses comparator groups were based on the Australia-wide population due to Queensland-specific data being unavailable. Fourth, although the cause-specific excess mortality by diagnostic groups are presented in the Supplementary Materials, the primary analyses aggregate heterogeneous conditions, and the pooled cohort estimates should be interpreted with the recognition that the underlying causal pathways to premature death differ markedly between, for example, substance use disorders and psychotic illness. Fifth, because each individual was assigned a single principal-diagnosis group using the severity-based hierarchy, psychiatric comorbidity is subsumed rather than examined. The effects of psychiatric comorbidities (e.g. substance use in people with schizophrenia), therefore, were not tested, and represent a direction for future research. Further, dedicated analyses of other mental disorders (e.g. eating disorders) was not conducted due to the low numbers of observed deaths. Further research with larger cohorts should aim to examine these smaller diagnostic groups. Finally, the underlying cause of death was coded as a single ICD-10-AM code, which does not capture the contribution of comorbid conditions to mortality. We could not compute population attributable fractions due to a lack of reliable prevalence estimates in the general population.

### Conclusion

The life expectancy gap between people with hospitalised psychiatric illness and the general population in Queensland has narrowed significantly over the past 14 years, driven primarily by improvements in survival within the psychiatric cohort. The gap, however, remains large in absolute terms, and people with schizophrenia and related psychotic disorders have not shared equally in these life expectancy gains. Sustained and targeted efforts to improve physical health monitoring, treatment access, and therapeutic innovation for this group remain essential.

## Contributors

Mike Trott, David Lawrence, Sean Halstead, Claudia Bull, Dan Siskind, and Steve Kisely conceptualised the study. Mike Trott curated the data and, with Steve Kisely, conducted the formal analysis. Mike Trott led the software, investigation, methodology and visualisation, with methodological input from David Lawrence and Sean Halstead and additional visualisation from Dan Siskind. Claudia Bull led project administration and Dan Siskind provided supervision. Steve Kisely validated the analysis. Mike Trott, Dan Siskind and Steve Kisely wrote the original draft, and all authors contributed to review and editing.

## Data sharing statement

Due to privacy, ethical and legal considerations, the administrative health data cannot be shared without direct approval from relevant data custodians and the Office of Research and Innovation of Queensland Health. Contact details for Queensland Health custodians can be found at https://www.health.qld.gov.au/__data/assets/pdf_file/0034/843199/data_custodian_list.pdf.

## Declaration of interests

No author has any financial or commercial interests relating to this study.
