# Supplementary material for: Change in the life expectancy gap among people with mental illness: a 14-year population-based cohort study in Australia
Source: Lancet Reg Health West Pac. 2026 Sep 18;74:101978. doi: 10.1016/j.lanwpc.2026.101978 (PMC13649517; doi:10.1016/j.lanwpc.2026.101978)
Supplement: Supplementary file 1 [file mmc1.docx]

**Supplementary Table 1**: ICD-10-AM codes for causes of death categories

| **Cause of death** | **ICD-10-AM codes** |
| --- | --- |
| Coronary heart disease | I20-I25 |
| Cerebrovascular disease | I60-I69 |
| All cancers | C00-D48 |
| COPD | J40-J44 |
| Influenza and pneumonia | J09-J18 |
| Diabetes | E10-E14 |
| Liver disease | K70-K76 |
| Chronic kidney disease | B52·0, D59·3, E10·2, E11·2, E12·2, E13·2, E14·2, E85·1, I12, I13, I15·0, I15·1, N00–N07, N11, N12, N14, N15, N18, N19, N25–N28, N39·1, N39·2, Q60–Q63, T82·4, T86·1 |
| Dementia (including Alzheimer’s) | F01,F03,G30 |
| Intentional self-harm (suicide) | X60-X84; Y87·0 |
| Accidental poisoning | X40-X49 |
| Assault | X85-Y09; Y87·1 |

Codes derived and matched to the codes used in the General Record of Incidence of Mortality (GRIM) books; COPD = Chronic Obstructive Pulmonary Disease

**Supplementary Table 2.** Cohort demographic characteristics by calendar period

| **Principal diagnosis** | | **2009–2011** | **2012–2014** | **2015–2017** | **2018–2020** | **2021–2023** |
| --- | --- | --- | --- | --- | --- | --- |
| Schizophrenia | n | 2,934 | 3,144 | 3,086 | 2,537 | 1,846 |
|  | Median age  (IQR) | 32  (23; 44) | 31  (23; 43) | 32  (23; 43) | 32  (24; 44) | 34  (25; 48) |
|  | Percent female | 36 | 35 | 32.8 | 33.9 | 36.6 |
|  | Median follow-up  (years) | 13.3 | 10.4 | 7.4 | 4.6 | 1.7 |
| Affective psychosis | n | 2,718 | 2,848 | 2,893 | 2,828 | 2,307 |
|  | Median age  (IQR) | 39  (27; 51) | 37  (27; 50) | 38  (26; 51) | 36  (25; 50) | 38  (27; 52) |
|  | Percent female | 60.9 | 60.2 | 57.8 | 58.2 | 57.7 |
|  | Median follow-up (years) | 13.3 | 10.4 | 7.5 | 4.5 | 1.6 |
| Other psychotic disorders | n | 1380 | 1703 | 2057 | 2486 | 2613 |
|  | Median age  (IQR) | 37  (26; 51) | 35  (25; 48) | 36  (26; 49) | 36  (25; 48) | 36  (25; 50) |
|  | Percent female | 49.1 | 46.7 | 45.4 | 46.9 | 49.9 |
|  | Median follow-up  (years) | 13.1 | 10.3 | 7.3 | 4.3 | 1.3 |
| Alcohol or substance use disorders | n | 8,689 | 10,721 | 11,953 | 13,428 | 12,135 |
|  | Median age  (IQR) | 37  (26; 49) | 35  (25; 47) | 35  (25; 48) | 36  (25; 48) | 37  (26; 50) |
|  | Percent female | 38.9 | 39.2 | 37.6 | 38 | 38 |
|  | Median follow-up (years) | 13.2 | 10.2 | 7.3 | 4.3 | 1.5 |
| Neurotic disorders | n | 4,173 | 5,607 | 6,755 | 7,946 | 7,754 |
|  | Median age  (IQR) | 48  (33; 65) | 46  (32; 63) | 45  (30; 63) | 42  (28; 60) | 41  (27; 60) |
|  | Percent female | 64.8 | 65.7 | 64.3 | 65 | 66.2 |
|  | Median follow-up  (years) | 13.1 | 10.2 | 7.2 | 4.3 | 1.6 |
| Stress and adjustment disorders | n | 4,223 | 4,947 | 4,836 | 5,176 | 5,337 |
|  | Median age  (IQR) | 38  (26; 50) | 37  (25; 48) | 38  (26; 50) | 38  (27; 51) | 39  (27; 51) |
|  | Percent female | 45.3 | 46.3 | 46.6 | 46.2 | 49.9 |
|  | Median follow-up  (years) | 13.3 | 10.3 | 7.3 | 4.4 | 1.5 |
| Depressive disorders | n | 4,756 | 5,716 | 7,255 | 6,560 | 6,914 |
|  | Median age  (IQR) | 44  (31; 57) | 43  (29; 57) | 42  (28; 56) | 41  (27; 56) | 43  (28; 60) |
|  | Percent female | 56.9 | 52.7 | 51.4 | 52 | 53.9 |
|  | Median follow-up  (years) | 13.2 | 10.2 | 7.3 | 4.4 | 1.5 |

**Supplementary Table 3.** Diagnostic composition of cohort entrants by calendar period

| **Principal diagnosis** | **2009–2011** | **2012–2014** | **2015–2017** | **2018–2020** | **2021–2023** |
| --- | --- | --- | --- | --- | --- |
| Schizophrenia | 2,934 (10·2) | 3,144 (9·1) | 3,086 (8·1) | 2,537 (6·1) | 1,846 (4·7) |
| Affective psychosis | 2,718 (9·4) | 2,848 (8·2) | 2,893 (7·6) | 2,828 (6·8) | 2,307 (5·9) |
| Other psychotic disorders | 1,380 (4·8) | 1,703 (4·9) | 2,057 (5·4) | 2,486 (6·0) | 2,613 (6·7) |
| Alcohol or substance use disorders | 8,689 (30·1) | 10,721 (30·9) | 11,953 (31·3) | 13,428 (32·2) | 12,135 (31·2) |
| Neurotic disorders | 4,173 (14·5) | 5,607 (16·2) | 6,755 (17·7) | 7,946 (19·1) | 7,754 (19·9) |
| Stress and adjustment disorders | 4,223 (14·6) | 4,947 (14·3) | 4,836 (12·7) | 5,176 (12·4) | 5,338 (13·7) |
| Depressive disorders | 4,756 (16·5) | 5,716 (16·5) | 6,560 (17·2) | 7,255 (17·4) | 6,914 (17·8) |

**Supplementary Table 4.** Diagnostic composition of person-time by calendar period

| **Principal diagnosis** | **2009–2011** | **2012–2014** | **2015–2017** | **2018–2020** | **2021–2023** |
| --- | --- | --- | --- | --- | --- |
| Schizophrenia | 17·6 | 15·6 | 14·0 | 12·6 | 11·2 |
| Affective psychosis | 10·6 | 10·3 | 9·7 | 9·2 | 8·7 |
| Other psychotic disorders | 5·2 | 5·2 | 5·2 | 5·3 | 5·5 |
| Alcohol or substance use disorders | 28·3 | 28·7 | 29·2 | 29·6 | 29·9 |
| Neurotic disorders | 10·6 | 11·7 | 12·8 | 13·8 | 14·8 |
| Stress and adjustment disorders | 13·6 | 14·0 | 14·0 | 13·9 | 13·9 |
| Depressive disorders | 14·1 | 14·6 | 15·1 | 15·6 | 16·0 |

**Supplementary Table 5:** Primary versus composition-standardised life expectancy gap, all psychiatric diagnoses combined

| **Sex** | **Estimate** | **2009–2011** | **2012–2014** | **2015–2017** | **2018–2020** | **2021–2023** |
| --- | --- | --- | --- | --- | --- | --- |
| Female | Primary analysis gap (years) | 13·4 | 12·3 | 11·3 | 10·3 | 9·4 |
|  | Composition-standardised gap (years) | 13·8 | 12·9 | 11·7 | 10·9 | 10·5 |
| Male | Primary analysis gap (years) | 16·7 | 15·3 | 14·5 | 12·8 | 12·2 |
|  | Composition-standardised gap (years) | 16·2 | 14·9 | 14·2 | 12·3 | 11·8 |

**Supplementary Table 6**: Observed, expected, and excess deaths by cause of death and principal psychiatric diagnosis, stratified by sex

| **Cause of death** | **Observed deaths** | **Expected deaths** | **Excess deaths** | **Mortality rate (per 100,000 PY)** | **Excess mortality rate (per 100,000 PY)** | **AFe (%)** |
| --- | --- | --- | --- | --- | --- | --- |
| Schizophrenia - Males | | | | | | |
| Intentional self-harm (suicide) | 319 | 49·2 | 269·8 | 162·2 | 137·2 | 84·6 |
| Coronary heart disease | 310 | 69·5 | 240·5 | 157·6 | 122·2 | 77·6 |
| Accidental poisoning | 193 | 25·9 | 167·1 | 98·1 | 84·9 | 86·6 |
| All cancers | 315 | 196·0 | 119·0 | 160·1 | 60·5 | 37·8 |
| COPD | 128 | 17·4 | 110·6 | 65·1 | 56·2 | 86·4 |
| Diabetes mellitus | 61 | 16·4 | 44·6 | 31·0 | 22·7 | 73·1 |
| Cerebrovascular disease | 49 | 18·5 | 30·5 | 24·9 | 15·5 | 62·2 |
| Liver disease | 47 | 20·1 | 26·9 | 23·9 | 13·7 | 57·3 |
| Influenza and pneumonia | 31 | 4·7 | 26·3 | 15·8 | 13·4 | 84·7 |
| Assault | 18 | 3·8 | 14·2 | 9·2 | 7·2 | 78·8 |
| Chronic kidney disease | 13 | 8·0 | 5·0 | 6·6 | 2·5 | 38·4 |
| Dementia incl. Alzheimer's | 13 | 8·0 | 5·0 | 6·6 | 2·5 | 38·2 |
| Schizophrenia - Females | | | | | | |
| Coronary heart disease | 146 | 22·4 | 123·6 | 144·1 | 122·0 | 84·6 |
| All cancers | 257 | 142·6 | 114·4 | 253·7 | 112·9 | 44·5 |
| Intentional self-harm (suicide) | 86 | 7·5 | 78·5 | 84·9 | 77·4 | 91·2 |
| COPD | 87 | 16·1 | 70·9 | 85·9 | 69·9 | 81·5 |
| Accidental poisoning | 71 | 5·1 | 65·9 | 70·1 | 65·0 | 92·8 |
| Cerebrovascular disease | 62 | 16·9 | 45·1 | 61·2 | 44·5 | 72·7 |
| Diabetes mellitus | 51 | 9·7 | 41·3 | 50·3 | 40·8 | 81·0 |
| Influenza and pneumonia | 21 | 3·4 | 17·6 | 20·7 | 17·4 | 83·8 |
| Dementia incl. Alzheimer's | 30 | 14·5 | 15·5 | 29·6 | 15·3 | 51·8 |
| Liver disease | 18 | 6·8 | 11·2 | 17·8 | 11·0 | 62·1 |
| Chronic kidney disease | 17 | 6·6 | 10·4 | 16·8 | 10·2 | 61·0 |
| Assault | 4 | 0·8 | 3·2 | 3·9 | 3·2 | 80·9 |
| Affective psychosis - Males | | | | | | |
| Intentional self-harm (suicide) | 190 | 20·2 | 169·8 | 226·3 | 202·2 | 89·3 |
| Coronary heart disease | 115 | 57·1 | 57·9 | 137·0 | 69·0 | 50·4 |
| Accidental poisoning | 57 | 9·6 | 47·4 | 67·9 | 56·4 | 83·1 |
| All cancers | 208 | 171·8 | 36·2 | 247·7 | 43·1 | 17·4 |
| COPD | 39 | 18·8 | 20·2 | 46·4 | 24·1 | 51·8 |
| Diabetes mellitus | 34 | 14·6 | 19·4 | 40·5 | 23·0 | 56·9 |
| Influenza and pneumonia | 16 | 4·1 | 11·9 | 19·1 | 14·2 | 74·4 |
| Cerebrovascular disease | 28 | 17·7 | 10·3 | 33·3 | 12·2 | 36·6 |
| Dementia incl. Alzheimer's | 21 | 11·8 | 9·2 | 25·0 | 11·0 | 43·9 |
| Chronic kidney disease | 11 | 7·9 | 3·1 | 13·1 | 3·7 | 28·1 |
| Liver disease | 13 | 12·3 | 0·7 | 15·5 | 0·8 | 5·5 |
| Assault | 2 | 1·5 | 0·5 | 2·4 | 0·7 | 27·3 |
| Affective psychosis - Females | | | | | | |
| Intentional self-harm (suicide) | 142 | 9·3 | 132·7 | 113·0 | 105·6 | 93·5 |
| Coronary heart disease | 128 | 31·5 | 96·5 | 101·9 | 76·8 | 75·4 |
| All cancers | 241 | 192·7 | 48·3 | 191·8 | 38·4 | 20·0 |
| Accidental poisoning | 51 | 6·2 | 44·8 | 40·6 | 35·6 | 87·8 |
| Cerebrovascular disease | 65 | 23·8 | 41·2 | 51·7 | 32·8 | 63·4 |
| COPD | 59 | 22·6 | 36·4 | 47·0 | 29·0 | 61·7 |
| Diabetes mellitus | 45 | 13·5 | 31·5 | 35·8 | 25·1 | 70·0 |
| Dementia incl. Alzheimer's | 48 | 21·1 | 26·9 | 38·2 | 21·4 | 56·1 |
| Influenza and pneumonia | 23 | 4·7 | 18·3 | 18·3 | 14·5 | 79·5 |
| Chronic kidney disease | 15 | 9·3 | 5·7 | 11·9 | 4·5 | 38·1 |
| Liver disease | 13 | 8·9 | 4·1 | 10·3 | 3·2 | 31·3 |
| Assault | 2 | 0·9 | 1·1 | 1·6 | 0·9 | 54·3 |
| Other psychotic disorders - Males | | | | | | |
| Intentional self-harm (suicide) | 143 | 15·2 | 127·8 | 231·5 | 206·9 | 89·4 |
| Coronary heart disease | 94 | 29·0 | 65·0 | 152·2 | 105·2 | 69·1 |
| All cancers | 130 | 84·9 | 45·1 | 210·4 | 73·0 | 34·7 |
| Accidental poisoning | 50 | 7·6 | 42·4 | 80·9 | 68·6 | 84·8 |
| Diabetes mellitus | 25 | 7·3 | 17·7 | 40·5 | 28·6 | 70·8 |
| COPD | 26 | 8·7 | 17·3 | 42·1 | 28·0 | 66·4 |
| Liver disease | 22 | 7·1 | 14·9 | 35·6 | 24·1 | 67·8 |
| Cerebrovascular disease | 21 | 8·7 | 12·3 | 34·0 | 20·0 | 58·8 |
| Dementia incl. Alzheimer's | 16 | 5·2 | 10·8 | 25·9 | 17·4 | 67·3 |
| Influenza and pneumonia | 8 | 2·1 | 5·9 | 12·9 | 9·6 | 74·1 |
| Assault | 7 | 1·1 | 5·9 | 11·3 | 9·5 | 84·0 |
| Chronic kidney disease | 1 | 3·8 | -2·8 | 1·6 | -4·6 | -283·7 |
| Other psychotic disorders - Females | | | | | | |
| Coronary heart disease | 68 | 12·6 | 55·4 | 123·4 | 100·5 | 81·4 |
| All cancers | 132 | 77·7 | 54·3 | 239·5 | 98·6 | 41·2 |
| Dementia incl. Alzheimer's | 60 | 8·5 | 51·5 | 108·9 | 93·4 | 85·8 |
| Intentional self-harm (suicide) | 37 | 4·1 | 32·9 | 67·1 | 59·8 | 89·0 |
| Cerebrovascular disease | 42 | 9·6 | 32·4 | 76·2 | 58·8 | 77·2 |
| Diabetes mellitus | 27 | 5·5 | 21·5 | 49·0 | 39·1 | 79·8 |
| Accidental poisoning | 22 | 2·7 | 19·3 | 39·9 | 35·0 | 87·7 |
| COPD | 23 | 9·0 | 14·0 | 41·7 | 25·3 | 60·7 |
| Liver disease | 16 | 3·7 | 12·3 | 29·0 | 22·3 | 76·7 |
| Influenza and pneumonia | 12 | 1·9 | 10·1 | 21·8 | 18·3 | 84·1 |
| Assault | 2 | 0·4 | 1·6 | 3·6 | 2·9 | 80·3 |
| Chronic kidney disease | 3 | 3·7 | -0·7 | 5·4 | -1·4 | -25·0 |
| Alcohol or substance use disorders - Males | | | | | | |
| Liver disease | 846 | 48·8 | 797·2 | 213·3 | 201·0 | 94·2 |
| Accidental poisoning | 660 | 47·6 | 612·4 | 166·4 | 154·4 | 92·8 |
| All cancers | 1184 | 616·7 | 567·3 | 298·5 | 143·0 | 47·9 |
| Intentional self-harm (suicide) | 628 | 96·7 | 531·3 | 158·3 | 134·0 | 84·6 |
| Coronary heart disease | 684 | 207·5 | 476·5 | 172·5 | 120·1 | 69·7 |
| COPD | 317 | 64·6 | 252·4 | 79·9 | 63·6 | 79·6 |
| Cerebrovascular disease | 203 | 62·4 | 140·6 | 51·2 | 35·4 | 69·3 |
| Diabetes mellitus | 149 | 52·7 | 96·3 | 37·6 | 24·3 | 64·6 |
| Dementia incl. Alzheimer's | 83 | 38·7 | 44·3 | 20·9 | 11·2 | 53·4 |
| Influenza and pneumonia | 58 | 14·8 | 43·2 | 14·6 | 10·9 | 74·5 |
| Assault | 47 | 7·1 | 39·9 | 11·9 | 10·1 | 84·9 |
| Chronic kidney disease | 39 | 27·8 | 11·2 | 9·8 | 2·8 | 28·7 |
| Alcohol or substance use disorders - Females | | | | | | |
| Liver disease | 418 | 14·3 | 403·7 | 167·6 | 161·8 | 96·6 |
| Intentional self-harm (suicide) | 260 | 18·9 | 241·1 | 104·2 | 96·7 | 92·7 |
| Accidental poisoning | 249 | 12·4 | 236·6 | 99·8 | 94·8 | 95·0 |
| All cancers | 475 | 264·7 | 210·3 | 190·4 | 84·3 | 44·3 |
| Coronary heart disease | 230 | 37·9 | 192·1 | 92·2 | 77·0 | 83·5 |
| COPD | 166 | 27·7 | 138·3 | 66·5 | 55·4 | 83·3 |
| Cerebrovascular disease | 117 | 28·6 | 88·4 | 46·9 | 35·4 | 75·5 |
| Dementia incl. Alzheimer's | 81 | 22·5 | 58·5 | 32·5 | 23·5 | 72·3 |
| Diabetes mellitus | 64 | 17·3 | 46·7 | 25·7 | 18·7 | 73·0 |
| Influenza and pneumonia | 40 | 6·0 | 34·0 | 16·0 | 13·6 | 85·1 |
| Assault | 12 | 1·9 | 10·1 | 4·8 | 4·1 | 84·4 |
| Chronic kidney disease | 21 | 11·5 | 9·5 | 8·4 | 3·8 | 45·3 |
| Neurotic disorders - Males | | | | | | |
| All cancers | 582 | 281·6 | 300·4 | 592·2 | 305·6 | 51·6 |
| Coronary heart disease | 275 | 92·7 | 182·3 | 279·8 | 185·5 | 66·3 |
| COPD | 184 | 34·8 | 149·2 | 187·2 | 151·9 | 81·1 |
| Intentional self-harm (suicide) | 136 | 22·6 | 113·4 | 138·4 | 115·4 | 83·4 |
| Cerebrovascular disease | 91 | 32·5 | 58·5 | 92·6 | 59·5 | 64·3 |
| Dementia incl. Alzheimer's | 76 | 27·0 | 49·0 | 77·3 | 49·9 | 64·5 |
| Diabetes mellitus | 67 | 25·2 | 41·8 | 68·2 | 42·5 | 62·3 |
| Accidental poisoning | 31 | 9·9 | 21·1 | 31·5 | 21·5 | 68·2 |
| Chronic kidney disease | 29 | 14·8 | 14·2 | 29·5 | 14·4 | 48·9 |
| Influenza and pneumonia | 20 | 7·4 | 12·6 | 20·4 | 12·8 | 63·0 |
| Liver disease | 28 | 15·9 | 12·1 | 28·5 | 12·3 | 43·1 |
| Assault | 5 | 1·5 | 3·5 | 5·1 | 3·5 | 69·5 |
| Neurotic disorders - Females | | | | | | |
| Coronary heart disease | 406 | 70·8 | 335·2 | 211·1 | 174·3 | 82·6 |
| All cancers | 665 | 363·8 | 301·2 | 345·8 | 156·7 | 45·3 |
| COPD | 289 | 49·5 | 239·5 | 150·3 | 124·5 | 82·9 |
| Cerebrovascular disease | 266 | 55·4 | 210·6 | 138·3 | 109·5 | 79·2 |
| Dementia incl. Alzheimer's | 229 | 59·6 | 169·4 | 119·1 | 88·1 | 74·0 |
| Intentional self-harm (suicide) | 108 | 13·3 | 94·7 | 56·2 | 49·3 | 87·7 |
| Diabetes mellitus | 113 | 29·3 | 83·7 | 58·8 | 43·5 | 74·1 |
| Influenza and pneumonia | 71 | 10·8 | 60·2 | 36·9 | 31·3 | 84·8 |
| Chronic kidney disease | 61 | 21·4 | 39·6 | 31·7 | 20·6 | 64·9 |
| Accidental poisoning | 36 | 8·4 | 27·6 | 18·7 | 14·3 | 76·6 |
| Liver disease | 26 | 14·5 | 11·5 | 13·5 | 6·0 | 44·4 |
| Assault | 5 | 1·3 | 3·7 | 2·6 | 1·9 | 74·6 |
| Stress and adjustment disorders - Males | | | | | | |
| Intentional self-harm (suicide) | 354 | 38·6 | 315·4 | 221·2 | 197·1 | 89·1 |
| Coronary heart disease | 187 | 102·5 | 84·5 | 116·9 | 52·8 | 45·2 |
| All cancers | 363 | 312·5 | 50·5 | 226·9 | 31·5 | 13·9 |
| COPD | 80 | 34·1 | 45·9 | 50·0 | 28·7 | 57·4 |
| Accidental poisoning | 64 | 18·5 | 45·5 | 40·0 | 28·4 | 71·1 |
| Diabetes mellitus | 63 | 26·7 | 36·3 | 39·4 | 22·7 | 57·6 |
| Liver disease | 40 | 22·7 | 17·3 | 25·0 | 10·8 | 43·2 |
| Dementia incl. Alzheimer's | 31 | 20·8 | 10·2 | 19·4 | 6·4 | 33·0 |
| Assault | 10 | 2·8 | 7·2 | 6·2 | 4·5 | 72·2 |
| Influenza and pneumonia | 12 | 7·3 | 4·7 | 7·5 | 2·9 | 39·3 |
| Cerebrovascular disease | 35 | 31·6 | 3·4 | 21·9 | 2·2 | 9·8 |
| Chronic kidney disease | 12 | 14·3 | -2·3 | 7·5 | -1·4 | -18·9 |
| Stress and adjustment disorders - Females | | | | | | |
| Intentional self-harm (suicide) | 127 | 11·1 | 115·9 | 86·8 | 79·3 | 91·3 |
| Coronary heart disease | 96 | 23·2 | 72·8 | 65·6 | 49·7 | 75·8 |
| All cancers | 211 | 162·4 | 48·6 | 144·2 | 33·2 | 23·0 |
| Accidental poisoning | 52 | 7·4 | 44·6 | 35·5 | 30·5 | 85·8 |
| COPD | 54 | 16·9 | 37·1 | 36·9 | 25·4 | 68·8 |
| Diabetes mellitus | 44 | 10·6 | 33·4 | 30·1 | 22·9 | 76·0 |
| Cerebrovascular disease | 45 | 17·5 | 27·5 | 30·8 | 18·8 | 61·1 |
| Dementia incl. Alzheimer's | 24 | 13·6 | 10·4 | 16·4 | 7·1 | 43·3 |
| Influenza and pneumonia | 12 | 3·6 | 8·4 | 8·2 | 5·7 | 69·7 |
| Liver disease | 17 | 8·8 | 8·2 | 11·6 | 5·6 | 48·2 |
| Chronic kidney disease | 13 | 7·0 | 6·0 | 8·9 | 4·1 | 46·2 |
| Assault | 1 | 1·1 | -0·1 | 0·7 | -0·1 | -9·3 |
| Depressive disorders - Males | | | | | | |
| Intentional self-harm (suicide) | 393 | 36·4 | 356·6 | 255·6 | 231·9 | 90·7 |
| All cancers | 547 | 366·2 | 180·8 | 355·7 | 117·6 | 33·0 |
| Coronary heart disease | 271 | 121·6 | 149·4 | 176·2 | 97·1 | 55·1 |
| COPD | 105 | 42·4 | 62·6 | 68·3 | 40·7 | 59·6 |
| Dementia incl. Alzheimer's | 91 | 29·9 | 61·1 | 59·2 | 39·7 | 67·1 |
| Cerebrovascular disease | 90 | 40·0 | 50·0 | 58·5 | 32·5 | 55·6 |
| Diabetes mellitus | 82 | 32·1 | 49·9 | 53·3 | 32·5 | 60·9 |
| Accidental poisoning | 44 | 16·6 | 27·4 | 28·6 | 17·8 | 62·3 |
| Influenza and pneumonia | 32 | 9·2 | 22·8 | 20·8 | 14·9 | 71·4 |
| Liver disease | 39 | 24·0 | 15·0 | 25·4 | 9·8 | 38·5 |
| Chronic kidney disease | 29 | 18·1 | 10·9 | 18·9 | 7·1 | 37·7 |
| Assault | 2 | 2·5 | -0·5 | 1·3 | -0·3 | -25·9 |
| Depressive disorders - Females | | | | | | |
| Coronary heart disease | 248 | 51·8 | 196·2 | 135·2 | 107·0 | 79·1 |
| Intentional self-harm (suicide) | 197 | 13·2 | 183·8 | 107·4 | 100·2 | 93·3 |
| All cancers | 458 | 293·5 | 164·5 | 249·7 | 89·7 | 35·9 |
| Cerebrovascular disease | 157 | 39·8 | 117·2 | 85·6 | 63·9 | 74·6 |
| Dementia incl. Alzheimer's | 146 | 38·9 | 107·1 | 79·6 | 58·4 | 73·4 |
| COPD | 96 | 36·7 | 59·3 | 52·3 | 32·3 | 61·8 |
| Diabetes mellitus | 65 | 21·9 | 43·1 | 35·4 | 23·5 | 66·3 |
| Accidental poisoning | 43 | 8·7 | 34·3 | 23·4 | 18·7 | 79·9 |
| Influenza and pneumonia | 42 | 7·8 | 34·2 | 22·9 | 18·6 | 81·3 |
| Liver disease | 37 | 13·0 | 24·0 | 20·2 | 13·1 | 64·8 |
| Chronic kidney disease | 31 | 15·5 | 15·5 | 16·9 | 8·5 | 50·1 |
| Assault | 5 | 1·3 | 3·7 | 2·7 | 2·0 | 74·4 |

AFe = attributable fraction

**Supplementary Table 7:** Temporal trends in cause-specific standardised mortality ratios, stratified by sex

| **Cause of death** | **Males** | | **Females** | |
| --- | --- | --- | --- | --- |
|  | **% change per period** | **p-value** | **% change per period** | **p-value** |
| All cancers | -5·9% | 0·008 | -4·0% | 0·032 |
| Assault | -8·1% | 0·225 | -22·3% | 0·022 |
| Cerebrovascular disease | -6·0% | 0·084 | -2·7% | 0·225 |
| Chronic kidney disease | -20·5% | 0·062 | -8·1% | 0·315 |
| Coronary heart disease | -6·4% | 0·012 | -1·4% | 0·540 |
| COPD | -11·4% | 0·018 | -6·3% | 0·079 |
| Dementia incl· Alzheimer's | -2·7% | 0·657 | +6·7% | 0·293 |
| Diabetes mellitus | -7·2% | 0·031 | -5·2% | 0·119 |
| Influenza and pneumonia | -5·8% | 0·116 | -9·0% | 0·029 |
| Intentional self-harm | -9·8% | 0·005 | -12·5% | 0·002 |
| Liver disease | -11·4% | 0·045 | -10·5% | 0·014 |

**Supplementary Figure 1**: Standardised mortality ratios by sex and principal diagnosis

**Supplementary Figure 2**: Proportions of excess deaths from pre-COVID-19 (solid colours) to post-COVID-19 (faded colours), stratified by cause of death and sex
